# Microscopic Analysis of Temperature Effects on Surface Colonization and Biofilm Morphology of *Salmonella enterica*

**DOI:** 10.3390/foods14020268

**Published:** 2025-01-15

**Authors:** Zachariah Vice, You Zhou, Sapna Chitlapilly Dass, Rong Wang

**Affiliations:** 1 Department of Animal Science, Texas A&M University, College Station, TX 77845, USA; 2 Center for Biotechnology, University of Nebraska–Lincoln, Lincoln, NE 68588, USA; 3 U.S. Meat Animal Research Center, Agriculture Research Service (ARS), U.S. Department of Agriculture (USDA), Clay Center, NE 68933, USA

**Keywords:** *Salmonella enterica*, biofilms, confocal microscopy, scanning electron microscopy

## Abstract

*Salmonella enterica* represents a diverse group of pathogens commonly associated with food contamination including red meat. Even though pre- and post-harvest cleaning and sanitization procedures are widely implemented at meat processing plants to mitigate the hazard, *S. enterica* cells may escape the process by colonizing, on contact, surfaces in the form of a biofilm that functions as an aggregated microbial community to facilitate mutual protection, antimicrobial resistance, proliferation and dissemination. Biofilm development is a complex process that can be affected by a variety of factors including environmental temperature. We developed methods using scanning electron microscopy and confocal microscopy with a novel image analysis software tool to investigate the temperature influence on *S. enterica* cell colonization and biofilm formation by directly visualizing and comparing the biofilm matrix’s morphological differences under various temperatures. Cocktails of *S. enterica* strains belonging to serovars, commonly isolated from meat samples, were applied to develop biofilms on a stainless steel surface at 7, 15, or 37 °C. Results of the microscopy analysis showed that as temperature increased, better-defined biofilm structures with extracellular polymeric structures (EPS) could be identified. However, *S. enterica* colonization and aggregated bacterial biomass were clearly observed at the low temperature (7 °C) as well. These results demonstrate that the environmental temperature significantly contributes to *S. enterica* biofilm formation as the higher temperatures encourage bacterial active proliferation and biofilm maturation leading to the development of well-pronounced structures, while the lower temperature may promote cell attachment but, meanwhile, limit the EPS biosynthesis and biofilm maturation. Our study indicates that the mature *S. enterica* biofilms formed under favorable conditions may protect the pathogens with the well-developed 3-demensional (3D) structure against routine treatment. Furthermore, the low temperatures commonly maintained at meat plants are not able to effectively prevent *S. enterica* colonization and biofilm formation since at such temperatures there could still be colonized biomass that can contaminate the products. Therefore, the temperature effect on pathogen colonization and biofilm development should be taken into consideration while evaluating hygiene standards and sanitization procedures at the processing facilities.

## 1. Introduction

Foodborne illness remains a significant public health concern worldwide. Despite the ever-growing understanding of such threats and advanced regulatory frameworks in place to combat the pathogenic microorganisms that cause diseases, people are still affected by foodborne infections and outbreaks across the globe. Among the foodborne pathogens most implicated in widespread outbreaks is *Salmonella enterica* [1]. In the United States, non-typhoidal *S. enterica* species were reported as the leading cause of hospitalization and death from gastrointestinal infections [2].

*S. enterica is* a Gram-negative bacterial species commonly associated with the contamination of various food types including beef products [3]. Cattle are known harborages for *Salmonella* spp. [4]. During beef slaughter and processing, contaminated effluent can contact various surfaces within the facility, resulting in the potential for future cross-contamination events. While post-harvest cleaning procedures are required under HACCP (Hazard Analysis and Critical Control Points) protocols which should mitigate the hazard, *S. enterica* cells may escape the cleaning and sanitation procedure and begin colonization on contact surfaces and proliferation in hard-to-clean areas within the processing facility in the form of a biofilm.

Biofilm development is a complex process that can be affected by a variety of factors. Bacterial strains’ unique intrinsic properties have a major influence, meanwhile, many environmental conditions such as temperature, pH, salinity, nutrient density, and interactions with other microorganisms in the environment could significantly impact *S. enterica* biofilm formation. Among the many environmental factors, the temperature influence on *S. enterica* biofilms has been tested over a wide range. Overall, most studies showed that higher temperatures would lead to more robust *S. enterica* biofilm development compared to the lower temperatures and such impact could also be strain-dependent and/or in combination with other factors such as nutrient availability and pH [5,6,7,8]. Furthermore, at the genomic level, different patterns of temperature effect on the expression of biofilm-producing genes, virulence genes, and stress genes of various *S. enterica* strains/serovars were also investigated. For instance, Roy et al. indicated that the optimum combination of environmental conditions (temperature 37 °C, pH 7.0, and 0.25% glucose) would promote strong *S. enterica* biofilm development on food contact surfaces and increase the virulence gene expression levels. Badie et al. evaluated the 2-factor influence of temperature (2, 8, 22.5, 37, 43 °C) and pH (2.4, 3, 4.5, 6, 6.6) over a wide range and showed that the *S*. Enteritidis biofilm-producing genes *adr A* and *bap A*, virulence genes *hil A* and *inv A*, and stress gene *RpoS* each reached its highest expression level under different but unique combination of the two factors. This study highlights the critical involvement of multiple environmental conditions in *S. enterica* survival and virulence.

Since *S. enterica* is a diverse group of pathogens with over 2600 serovars, it is well expected that the unique intrinsic properties of the various *S. enterica* strains and serovars would vary significantly, and this vast pathogenic group would apply and adapt multiple strategies for survival, thus, express a wide variety of phenotypes including biofilm formation under different conditions. Low temperatures are usually maintained in most areas of the meat processing plants to prevent bacterial outgrowth. However, bacterial colonization and biofilm development by *S. enterica* strains/serovars under low temperatures were investigated previously. A range of observations has been reported, including some seemingly conflicting results, which could likely be attributed to the inherent variability among the diverse serovars/strains utilized in the studies [5,7,8,9,10,11].

Therefore, to understand the potential contribution of biofilm formation to *S. enterica* persistence at meat plants, we used *S. enterica* strains isolated from meat processing facilities and beef trim to investigate the temperature influence on *S. enterica* cell colonization and biofilm development by directly visualizing and, with the aid of novel software tools, analyzing the morphological differences of *S. enterica* biofilm matrix on common food contact surface under different temperatures. The objective of the study is to understand how the temperature factor affects these microorganisms’ phenotypic and morphological expressions at their biofilm stage. Since these strains were obtained directly from the meat processing environment, their actual ability to attach to the contact surface and form biofilms under the current industry settings could significantly contribute to their survival and persistence in the plants. Their biofilm morphological variations related to environmental changes (i.e., temperature) would allow us to better understand how such pathogenic biofilms adapt to favorable or unfavorable conditions. Such information could help develop more niche-specific intervention strategies to disrupt and prevent biofilms at the various development stages under different conditions.

## 2. Materials and Methods

### 2.1. Revival and Preparation of S. enterica Strains

*S. enterica* isolates were obtained from the Microbial Ecology and Microbiome Interaction Laboratory (Department of Animal Science, Texas A&M University, College Station, TX, USA), and the U.S Meat Animal Research Center (ARS, USDA, Clay Center, NE, USA). Isolates of *S. enterica* subsp. serovar Anatum, Dublin, Montevideo, Newport, and Typhimurium in glycerol (15%) suspensions were thawed from −80 °C storage. Upon thawing, 200 µL of thawed culture was transferred to 10 mL of sterile lysogeny broth without salt (LB-NS; Thermo Fisher Scientific, Waltham, MA, USA) per isolate, and incubated at 37 °C overnight. Isolates were then streak-plated onto Xylose-Lysine Deoxycholate Agar (HiMedia Laboratories LLC, Kennett Square, PA, USA) plates and incubated 24 h at 37 °C. Well-isolated black colonies were picked, streaked onto Nutrient Agar (Ward’s Science, Rochester, NY, USA), and incubated for 24 h at 37 °C. Finally, well-defined colonies were picked and streaked onto nutrient agar slants and incubated at 37 °C for 24 h. After incubation, isolates were stored at 7 °C for no longer than 7 days to facilitate further experiments.

### 2.2. Inoculum Preparation

To develop dual-strain biofilms for confocal microscope analysis, 10 µL loops of each *S. enterica* strain (serovars Typhimurium and Montevideo) were pulled and used to co-inoculate a 10 mL tube of sterile LB-NS. The inoculated LB-NS was then incubated for 18–24 h at 37 °C then centrifuged for 15 min at 2209 times gravity (× g). Following centrifugation, the supernatant was gently removed and 5 mL of sterile LB-NS was added. The pellet was vortexed to resuspend cells in each tube and then the *S*. Typhimurium and *S*. Montevideo strains were combined, rendering a 10 mL cocktail containing both microorganisms. The mixture was vortexed again to ensure homogenous distribution of the microorganisms, then centrifuged at 2209× *g* for 15 min. The supernatant was removed and 10 mL of sterile LB-NS was added to resuspend the pellet by vortexing.

### 2.3. Biofilm Development, Staining, and Confocal Microscope Imaging

Sterile stainless steel (SS) coupons (2 × 2 × 0.3 cm) were aseptically placed into individual wells of a six-well plate (Corning Costar™, Fisher Scientific, Corning, NY, USA). For inoculation, an *S. enterica* cocktail inoculum (5.0 × 10^8^ CFU/500 µL) was carefully dispensed onto the top of each coupon, ensuring that no fluid ran off the edges into the surrounding well. One of the wells was filled with 10 mL of distilled water to provide residual moisture to the microorganisms during incubation. The plate was covered with the lid and incubated for five days at 7 °C, 15 °C, or 37 °C.

After incubation at the respective temperatures, SS coupons were lifted using sterile forceps and tilted to allow excess media or unattached cells to run off the edge of the coupon. Invitrogen™ FM™ 1-43 Dye (N-(3-Triethylammoniumpropyl)-4-(4-(Dibutylamino) Styryl) Pyridinium Dibromide) (Thermo Fisher Scientific, Eugene, OR, USA) was 1:1000 diluted and a 500 µL staining fluid aliquot was carefully added onto the top of the biofilm on the coupon as not to disrupt the biofilm layers. The coupons were then placed in the six-well plate, covered with foil and allowed to incubate at room temperature for 15 min. After incubation, coupons were lifted using sterile forceps and tilted to allow excess dye to run off the edge, then placed on a paper towel to remove any excess moisture from the underside.

Glass microscopy slides were laid on paper towels and a small amount of nail polish was dispensed at the middle of the slide. The coupons were then placed, biofilm-side up, onto the nail polish of the microscopy slide. Using the sterile forceps, the edges of the coupons were carefully pressed down to distribute the nail polish underneath to ensure levelness. The biofilm was then overlaid with a square (18 mm × 18 mm), #1.5 glass coverslip (Corning Inc., Corning, NY, USA). The prepared samples were then analyzed with a Zeiss LSM 780 inverted confocal microscope (Carl Zeiss AG, Oberkochen, Germany) and a 488 nm laser line was used for excitation of the target dye (FM™ 1-43). Biofilms developed at each temperature were imaged and analyzed separately. Using the inverted confocal microscope which was equipped with a plan-apochromat 20×/0.8 NA objective and a 1.5× optical zoom, using a 3 × 3-frame ‘tile’ function and a Z-stack.

### 2.4. Image Cytometry of Biofilm Confocal Images

The “Z-stack”.czi files produced by ZEN imaging software (Version 2.3, Blue Edition, Carl Zeiss AG, Oberkochen, Germany) using confocal microscopy were imported into BiofilmQ [12] and the obtained large micrographs were each divided into four sub-Z-stacks as individual subsamples that retained the original numbers of slices and layers. BiofilmQ was operated using MATLAB 2024a (Version 24.1.0.2537033, MathWorks, Inc., Natick, MA, USA) using the Texas A&M High-Performance Research Computing cluster “Grace”. Imported files were automatically converted into proprietary three-dimensional stacked-TIFF files for further processing by BiofilmQ. The image preparation process included colony separation (via semi-automatic input) and alignment (via means square regression). Subsequently, cropping, pre-processing, thresholding (Otsu method), and post-processing image polishing via the “3D Median of binary image” option were completed before segmentation. Finally, segmentation was performed using the “cubes” dissection method to allow for downstream parameter calculations.

The post-segmentation parameters estimated by BiofilmQ included biofilm base area, height, mean thickness, volume, and roughness. Biofilm Mean Thickness (reported as “thickness”) was calculated as the mean value of all z-values for each base pixel of “pillars” (objects that share the same x and y coordinates) across the biofilm. The Architecture_LocalDensity_range30 mean biovolume was calculated as the occupied volume fraction of each “sphere” which was created by BiofilmQ with a radius of 30 µm around each object with “CentroidCoordinates” of x, y, and z. Each subsample was analyzed via BiofilmQ and the respective measurements were calculated. Means and standard errors of the mean (SEM) were analyzed using ANOVA followed by post-Turkey’s multiple comparisons tests. *p*-values lower than 0.05 were considered statistically significant.

To produce biofilm 3D images, .vtk files were imported into ParaView (Version: 5.13.0-RC1; Kitware, Inc., Clifton Park, NY, USA) and 3D graphics were generated based on the parameters calculated by BiofilmQ, namely “thickness” (Biofilm Mean Thickness) and “density” (Architecture_LocalDensity_range30 Mean Biovolume).

### 2.5. Scanning Electron Microscopy (SEM)

To evaluate *Salmonella* biofilm morphology using SEM, an *S. enterica* cocktail was prepared as described above by mixing equal volumes of overnight cultures of five *S. enterica* strains of various serovars (Anatum, Dublin, Montevideo, Newport, Typhimurium) that were isolated from beef trim samples [11]. The *S. enterica* cocktail was used for biofilm development on the SS surface by incubating the SS chips (18 × 18 × 2 mm, 2B brushed finish) in the prepared *S. enterica* cocktail for 5 days at 7 °C or 15 °C. The SS chips were rinsed with sterile water and then fixed with 2.5% glutaraldehyde in 0.1 M cacodylate buffer for 2 h at room temperature. Samples were rinsed again briefly and post-fixed with 1% osmium tetroxide for 1 h then processed for dehydration through an ethanol series (30 to 100%) and air dried. For SEM imaging, the chips were mounted onto the SEM stubs, placed in a 42 °C vacuum oven overnight, and sputter coated with a thin layer (8- to 10-nm thick) of chromium with a sputter coater (Desk V, Denton Vacuum, Moorestown, NJ, USA). The coated samples were examined under a field-emission scanning electron microscope (S-4700, Hitachi, Tokyo, Japan) to directly observe and evaluate the temperature effect on *S. enterica* biofilm structure and morphology.

### 2.6. Inoculum Quantification

To quantify the *S. enterica* cells inoculated for the experiment, each inoculum of the *S. enterica* cocktails was 10-fold diluted in phosphate-buffered saline (EMD Millipore Corp., Billerica, MA) and appropriate dilutions were plated onto 3M™ Aerobic Count Petrifilms™ (AC; 3M Company, Saint Paul, MN, USA) and incubated for 18–24 h at 37 °C. After incubation, the films were quantified using a 3M™ Petrifilm™ Plate Advanced Reader and a computer equipped with 3M™ Petrifilm™ Plate Manager software (Version 3.0.3). Quantification of each cocktail indicated approximately 10^9^ ± 0.1 CFU/mL of the *S. enterica* cells.

## 3. Results and Discussion

Confocal microscopy is a valuable research tool for bacterial biofilm studies by providing a detailed and comprehensive view of biofilm structure, composition, and dynamics. In the present study, the morphological structures of *S. enterica* biofilms grown at different temperatures were analyzed using confocal microscopy. All samples were inoculated with an identical concentration of 10^9^ ± 0.1 CFU/mL *S. enterica* cells based on the inoculum quantification.

**Figure 1:**
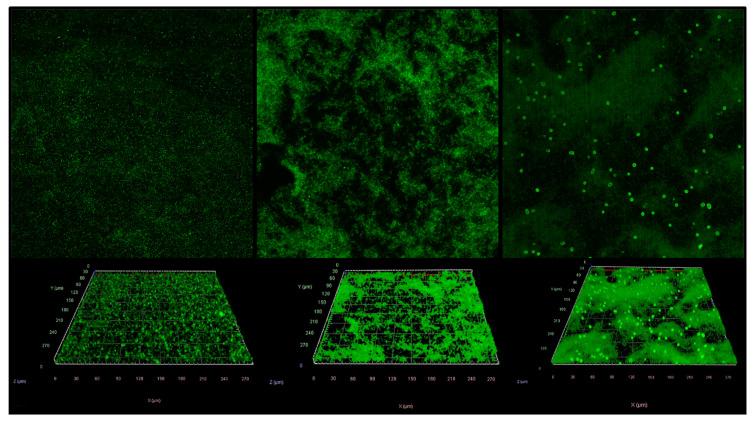
*S. enterica* biofilms formed at various temperatures are shown as confocal, single-exposure images (**top panel**) or three-dimensional projections of “Z-stacked” images (**bottom panel**). From left to right in each panel: biofilms formed at 7 °C, 15 °C, or 37 °C.

Z-stacks of the different temperature conditions were processed and compared (Figure 1). While the depth of the Z-stack and the number of “slices” varied among images due to optimal resolution differences among samples, visually substantial morphological differences among samples incubated at the various temperatures were observed. The samples incubated at 7 °C exhibited apparent cell attachment on the SS surface, which is consistent with our previous study that used single-strain cultures of 88 *S. enterica* strains belonging to 28 different *S. enterica* serovars. The bacterial enumeration results revealed a significant amount of *S. enterica* cell colonization on SS and PVC surfaces at 7 °C, ranging from 2.7 to 5.9 log_10_ CFU/cm^2^ [11]. However, the confocal microscope analysis in the present study showed that at 7 °C, the aggregations of *S. enterica* cells were sparse and not clearly distinguished from discrete, sessile cells. The colonized cells could be characterized by a lack of consistent structure organization across the field of view. Furthermore, the development of extracellular polymeric structures (EPS) was limited and not widespread.

Conversely, samples incubated at 15 °C exhibited organized biofilm matrix structures compared to those at 7 °C. More cellular aggregations were observed throughout the field of view (Figure 1), even though the distribution of such cellular aggregations appeared to be relatively random and did not follow a consistent pattern. Furthermore, there appeared to be obvious indications of EPS structures associated with the cellular aggregations, even though the distribution pattern of such structures was not consistent.

As the incubation temperature increased while all other environmental conditions remained constant, mature biofilms with more consistent distribution patterns and better- organized morphological structures were observed. The *S. enterica* cells incubated at 37 °C had widely spread cellular aggregations and consistent patterns of mature biofilm structure with enhanced presence of interstitial voids, which was in agreement with previous observations [13]. Notably, image analysis of biofilms developed at 37 °C also exhibited bright fluorescent signals approximately 2–5 µm in width/length evenly distributed across the field of view, which likely represented the individual or aggregated bacterium incorporated within the EPS matrix that was not observed in images of biofilms developed under the lower temperatures.

**Figure 2:**
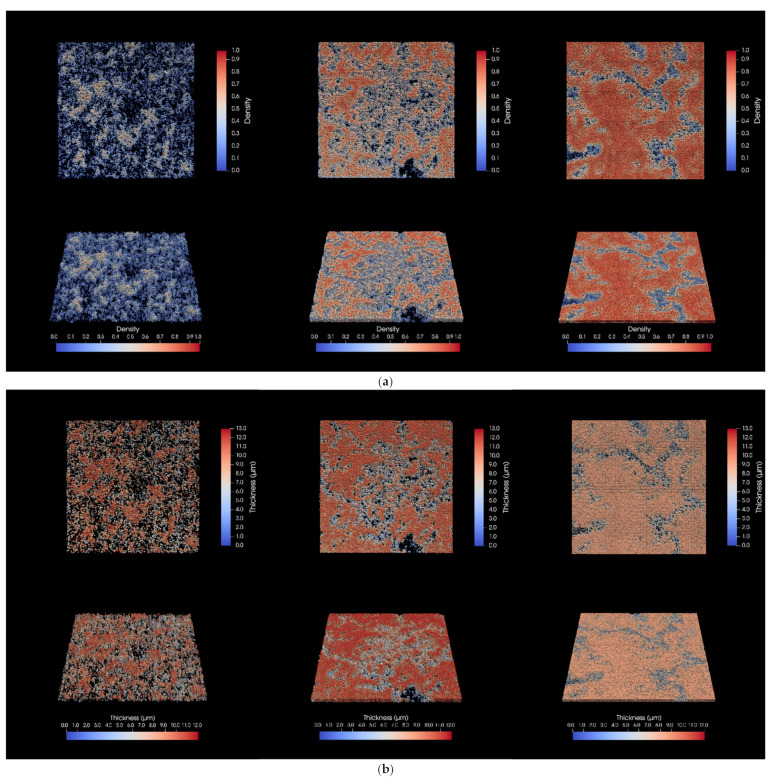
*S. enterica* biofilm 3D graphics panels generated by ParaView based on parameters calculated by BiofilmQ using 3D-visualization files (.vtk), showing biofilm structural density (**a**) and thickness (**b**). From left to right in each panel: biofilms formed at 7 °C, 15 °C, or 37 °C. The spectrum in color within the graphics indicates changes in density and biofilm thickness in (**a**,**b**), respectively.

BiofilmQ (Version 1.0.1) is a novel computational software tool that was developed and validated for analyzing fluorescence images of biofilm internal properties of the 3D spatial structures and performing data analysis/visualization with its integrated algorithms [12]. This software has been applied, with reliability and effectiveness, in the research field such as oral polymicrobial biofilm study [14] and spatial transcriptional heterogeneity comparison of *Bacillus subtilis* biofilms [15]. In the present study, this software tool was applied to generate the thickness and density panels of biofilm images with ParaView using the .vtk files (Figure 2). The 3D graphics more explicitly exhibited that the *S. enterica* biofilms had distinct and unique morphological features under each incubation temperature. At 7 °C, the biofilm demonstrated limited bacterial aggregation and few organized structures characteristic of biofilm development on the chip surface. At 15 °C, the *S. enterica* cell aggregates began to merge and form microcolonies. However, despite the increased biomatrix, the interstitial spaces between microcolonies still suggested limited EPS production. Conversely, *S. enterica* cells incubated at 37 °C exhibited a much more pronounced and organized structure with the highest density, indicating a well-developed, mature-stage biofilm.

The various parameters calculated by BiofilmQ for the biofilms developed under each temperature are shown in Table 1. No significant difference in biofilm base area was detected. Conversely, biofilm roughness and mean density both increased with the incubation temperatures, which is consistent with the 3D graphics showing that the density and maturity of the *Salmonella* biofilms progressively improved with the higher temperatures. Interestingly, there was a negative correlation in biofilm height and thickness as temperature increased, and biofilm volume, however, peaked at 15 °C. A relevant observation was reported in a previous study [16] investigating the progression of *S. enterica* biofilm development over an extended growth period (up to 144 h at 37 °C). This study detected the largest biovolume of live cells in biofilms at 72 h, then decreased with prolongated incubation, likely due to cell death and bacteria detachment prior to the next phase of colonization. While our present study focused on the temperature effect on the colonization and morphology of *S. enterica* biofilms, comparable mechanisms may underlie the observed similarities in the changing patterns of biofilm volume and the subsequent height/thickness across the temperature range or incubation period as both factors are likely to influence cell death and detachment in analogous ways and to a comparable extent.

**Figure 3:**
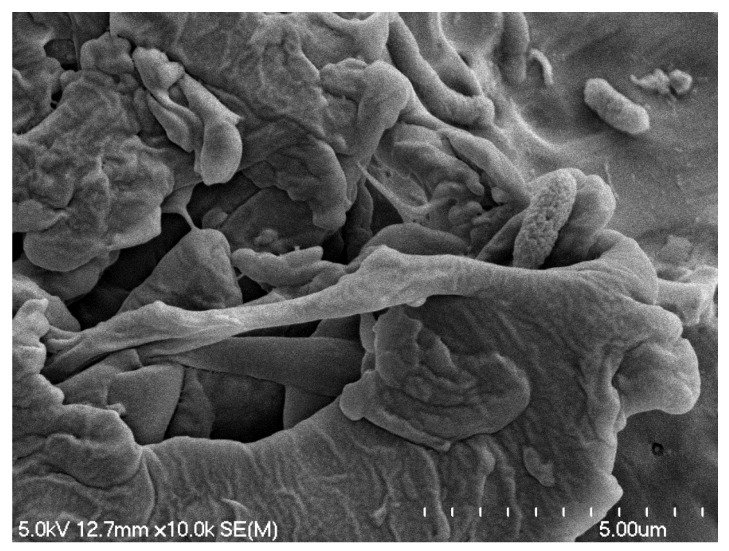
Representative SEM micrograph of biofilms formed by *S. enterica* cocktail strains at 7 °C on SS surface treated with sterile water. Magnification 10.0 k.

**Figure 4:**
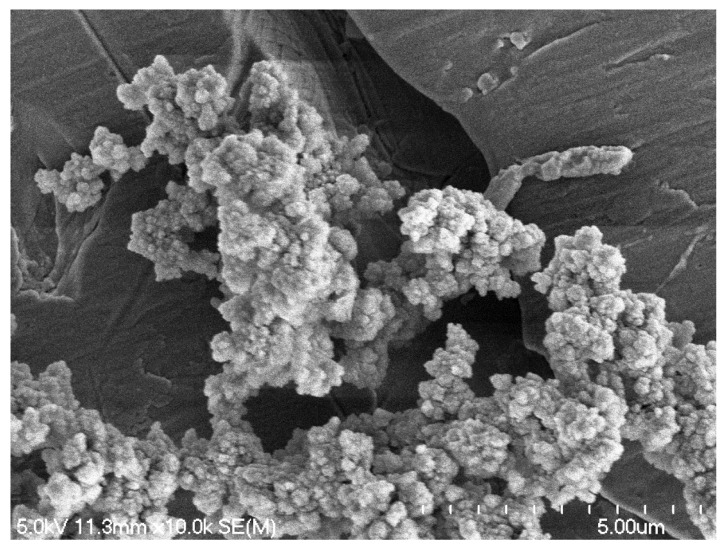
Representative SEM micrograph of biofilms formed by *S. enterica* cocktail strains at 15 °C on SS surface treated with sterile water. Magnification 10.0 k.

SEM is another powerful research tool for biofilm study that can provide detailed 3D images at high magnification, enabling direct visualization of the intricate architectures, including bacterial cells, EPS and other fine components within the biofilms and their spatial organization. To further closely visualize the biofilm matrix and the associated EPS expressions, SEM was applied in this study and the temperature effect on the architecture and cell surface structures of the *S. enterica* biofilms was also noticeable with such analysis. While the *S. enterica* biofilms developed by the strain cocktail were analyzed after 5 days of incubation at 7 °C or 15 °C, the *S. enterica* cells were observed to be able to colonize the SS surface and form microcolonies and biofilm structures under either temperature condition, which was consistent with the above observations using confocal microscopy. However, the environmental temperature affected biofilm morphology and EPS expression at different levels. *S. enterica* cells colonized under 7 °C exhibited more of the “flat smooth sheets” structure surrounded by thin layers of extracellular matrix, or clusters of bacteria as well as individually scattered cells with EPS expressed at various levels. The typical “cauliflower” shape with well-developed cell surface EPS that suggests a mature biofilm status was not widely distributed but could also be observed (Figure 3). Conversely, biofilms developed at 15 °C mostly exhibited a more stable and mature conformational characteristic compared to those at 7 °C, and the intensive three-dimensional “cauliflower” architectures well connected the bacteria with the SS surfaces by the strong production of the cell surface EPS structures (Figure 4).

Biofilm formation on contact surfaces is a bacterial survival strategy under adverse conditions which is a complex process generally involving five major steps: reversible non-specific attachment, irreversible attachment to the surface with bacterial adhesins, bacterial EPS production, biofilm maturation involving microcolony formation and signal molecular secretion, and finally, the detachment/dissemination stage. After the initial attachment, the maturation of the biofilm can be affected by a variety of factors including not only temperature but also nutrient availability, pH, moisture level, and the types of materials of the contact surface, etc. At this stage, the biomass of the initial aggregation of cells would replicate and express EPS structures and other appendages such as pili and fimbriae, which would lead to irreversible attachment and further aggregation of the biomass. Juliana et al. [17] developed models using binary responses to investigate the adhesion and biofilm development on stainless steel surfaces by various strains belonging to different *S. enterica* serovars and showed that the capability of *S. enterica* colonization/biofilm formation varied with strains/serovars as well as the different binary combinations of the experimental conditions including pH, NaCl, and a wide temperature range between 8 °C and 35 °C. Nitin et al. [18] also reported the combined influence of nutrients and temperature (10–22 °C) on *S. enterica* biofilm development.

The significant impact of environmental temperature on biofilm maturation and morphology has been investigated and results similar to our above observation have been reported [7,19,20]. For instance, Melo et al. [7] reported that using *S. enterica* serovar Minnesota strains, mature biofilm structure was not observed at 4 °C with SEM but instead only the biomass of punctual microcolonies was detected in the field. This study showed that biofilm formed at such low temperature was of low intensity, suggesting an initial stage of biofilm attachment or a potential stagnation at the early stage, whereas higher temperatures (25 and 36 °C) led to the development of dense bacterial clusters with extra matrix production, which is consistent with our present study.

The various *S. enterica* biofilm morphologies observed in the present and the previous studies are likely due to cell growth rates under different temperatures. *S. enterica* active replication was limited under 7 °C as we observed previously [21], which was also reported by other studies [5,7,8,22]. The adverse environmental conditions such as low temperature would lead to cell attachment but limit cell active replication and the biosynthesis of biofilm-related factors such as extracellular proteins and polysaccharides, which in turn, affect the biofilm maturation step with fully developed EPS structures. In the meat industry, low temperatures are usually maintained at most processing and storage areas at the plants to prevent bacterial outgrowth. However, our present study demonstrated that the low temperatures are not able to effectively prevent *S. enterica* surface attachment and colonization. This indicates a potential concern which was in agreement with a previous study [7] that even at 4 °C, surface colonized *S. enterica* biomass could be observed and might cause product contamination.

Furthermore, meat-processing plants may harbor a wide variety of microorganisms persisting in the environment as mixed biofilms. Foodborne pathogens, if present, are able to establish themselves in the multispecies communities to obtain higher stress tolerance and survival capability. For instance, our recent study showed that even though the *S. enterica* strains exhibited minimal growth (no significant cell density change) at 7 °C over the course of 5 days, the *S. enterica* strains were able to colonize within environmental mixed biofilms efficiently under such low temperature [23]. Based on such observation, we further applied differential staining fluorescence microscopy (DSFM) to track the location of the colonized *S. enterica* cells. This DSFM study showed that the *S. enterica* cells were evenly distributed through the upper half of the mixed biofilm structure, and the recruitment of the *S. enterica* strains into the multispecies community resulted in diverse alterations of the total biovolume of the mixed biofilms [21]. Therefore, besides persisting as a colonized single-species pathogen in the environment, *S. enterica* is also able to join the natural microbial communities and since multispecies biofilms are commonly seen in meat plants, such pathogen recruitment under low temperatures poses a real threat to meat safety.

It is noteworthy that the present study focuses only on the temperature factor while a variety of other environmental factors may potentially affect *S. enterica* biofilm maturation and morphology as discussed above and therefore warrants further investigation. In addition, contact surface materials play a critical role in initiating the process of cell attachment and biofilm formation by attracting bacterial cells to the target surface as a function of hydrophilic or hydrophobic repulsions/attractions. Stainless steel applied in our study is one of the most common materials used in the meat industry and its surface texture has been considered a favorable site for bacterial colonization and biofilm formation. However, other materials commonly applied in meat processing plants are worthy to be examined as well.

In conclusion, the present study demonstrated a direct correlation between the environmental temperature at which *S. enterica* biofilms developed and the organization/distribution of the microorganisms as well as the EPS expression. These results showed that the temperature factor significantly contributes to the biofilm morphology differences as the higher temperature encourages bacterial active growth and biofilm maturation leading to the development of well-pronounced structures, while the lower temperature may promote cell attachment but meanwhile inhibit cell replication and limit the EPS biosynthesis/expression and biofilm maturation. The strong physical architecture with the well-expressed cell surface structures may shelter the cells and block the penetration of the disinfectants, thus, effectively protecting the bacteria from chemical and physical stress. Meanwhile, our observations indicated that *S. enterica* can colonize and develop biomass under a wide temperature range and under low temperatures there still could be colonized biomass that can contaminate meat products. Therefore, the temperature effect on pathogen colonization and biofilm morphology should be taken into consideration while evaluating meat plant hygiene protocols, and sanitization procedures established at the processing facilities should be assessed on a case-to-case basis.

## Figures and Tables

**Table 1:** Parameters of representative *S. enterica* biofilm images calculated by BiofilmQ measuring biofilm size, base area, height, mean thickness, biofilm volume, roughness, and mean density in proportion. Data are shown as mean (SEM), n = 4. Values in the same column labeled with different superscript letters are statistically different (*p* < 0.05).

Temp. (°C)	X (µm)	Y (µm)	Z (µm)	Biofilm Base Area (µm^2^)	Biofilm Height (µm)	Biofilm Mean Thickness (µm)	Biofilm Volume (µm^3^)	Biofilm Roughness (µm)	Biofilm Mean Density (Proportion)
7	141.7	141.7	12.64	27,145.95 (1199.31)	9.76 (0.03) ^a^	7.88 (0.05) ^d^	9779.33 (511.54) ^g^	0.22 (0.0) ^j^	0.11 (0.0) ^l^
15	141.7	141.7	10.95	27,461.83 (275.88)	8.20 (0.05) ^b^	7.11 (0.15) ^e^	14,287.66 (1121.12) ^h^	0.23 (0.01) ^j^	0.14 (0.01) ^lm^
37	141.7	141.7	8.43	252,56.18 (1359.70)	6.12 (0.12) ^c^	5.00 (0.20) ^f^	2803.62 (382.48) ^i^	0.36 (0.02) ^k^	0.19 (0.02) ^m^

## Data Availability

The original contributions presented in this study are included in the article. Further inquiries can be directed to the corresponding authors.
